# What makes for effective, sustainable youth engagement in knowledge mobilization? A perspective for health services

**DOI:** 10.1111/hex.12918

**Published:** 2019-06-20

**Authors:** Eugenia Canas, Lisa Lachance, David Phipps, Cherrilyn Che Birchwood

**Affiliations:** ^1^ Western University London Ontario Canada; ^2^ Wisdom2Action Faculty of Health Dalhousie University Halifax Nova Scotia Canada; ^3^ York University Toronto Ontario Canada; ^4^ Université de Montréal Montreal Quebec Canada

**Keywords:** knowledge mobilization, mental health sector, service delivery, youth, youth engagement

## Abstract

**Background:**

Young people who seek mental health treatment often also seek the services of non‐profit organizations to support their well‐being. Wisdom2Action (W2A) is a Canadian knowledge mobilization network that focuses on improving the mental health and well‐being of children and youth in challenging contexts by increasing the use of evaluation, evidence and engagement in the youth‐serving sector. Since 2013, W2A has engaged youth advisors (YAs) to provide input to W2A's Board, lead their own projects and co‐design W2A activities.

**Objective:**

In fall 2017, the YAs, as well as adult Board members and W2A staff, collaborated in a participatory evaluation to better understand the experiences and impacts of youth engagement. This article describes insights derived through this process.

**Design and participants:**

Board, YAs and staff members participated in a reflective approach to informing, analysing and sharing the findings from this process. Individual interviews and review of documents, as well as iterative cycles of group analysis and synthesis, were conducted.

**Results:**

Both YAC members and W2A benefit from YAs’ leadership and engagement. The YAs position themselves as members of the youth‐serving sector, not merely recipients of its services; as such, their professional development aligns with the mandates of W2A and merits further investment, despite challenges in impact measurement.

**Conclusion:**

This article identifies challenges and facilitators of implementing an effective and sustainable youth advisory council model of engagement in the context of a pan‐Canadian network. The mutual gains and areas of growth for youth, adults and the organization described can inform health services, as well as funders and advocates for youth well‐being.

## 1 INTRODUCTION

Current youth engagement (YE) in mental health treatment planning,^1^ service development,^2^ research^3^, ^4^ and knowledge mobilization (KMb) has contributed to a developing understanding of the benefits and challenges of involving young people in the design of services and to increased calls for evaluation of such processes.^5^ In the youth‐serving sector, youth advisory councils (YAC) are an increasingly common feature of large‐scale research and KMb efforts, based on the principle that sustained youth perspectives are a necessary knowledge input into service and system improvement.^6^, ^7^

Wisdom2Action (W2A) is a Canada‐wide KMb network (Network) that focuses on improving mental health services provided to youth by improving the uptake of evidence and evaluation in the youth‐serving sector. W2A has also advocated for recognition of the role not‐for‐profit organizations (NPOs) can play in the well‐being of young people. Young people who seek mental health treatment often also seek the services of NPOs to support their material, spiritual, mental and physical well‐being. NPOs are particularly important for marginalized young people. Scholars note that young persons experiencing marginalization are more likely to use multiple services, need support for complex needs and have higher service‐use patterns for basic needs, such as education, employment and housing.^8^

Community‐based NPOs that provide services and programmes for youth rarely access evidence‐based practices (EBPs).^9^ In the past, NPOs and their programmes or services have not been included as part of mental health treatment planning, yet their services can be essential for a young person's well‐being.

W2A holds a history of investment in YE as part of its KMb activities to support NPOs. YE is a driving organizational value and a core component of Network processes and events. In this article, we reflect on the role of W2A's YAC, detailing its history, development and current significance upon the activities of the organization. Youth advisors (YAs), staff and Board members of W2A, including the authors, collaboratively evaluated the effectiveness and impact of this type of YE in relation to the Network's mission to strengthen the youth‐serving sector. Implications for KMb efforts in health services and research are included.

## 2 BACKGROUND

### 2.1 Knowledge mobilization

Knowledge mobilization is defined as the reciprocal and complementary flow and uptake of research between researchers, knowledge brokers and knowledge users, with the purpose of informing public debate, policies and/or practice, and improving services.^10^ KMb entails processes of knowledge generation, intra‐ and inter‐organizational interactions, and knowledge sharing. In addition to advocating for diverse forms of evidence to inform service and policy decision making, KMb can be conceptualized as a suite of activities and services that support the multidirectional connection of researchers with decision‐makers.^11^

The relationship between the sharing and mobilization of knowledge and the engagement of various stakeholders (including youth) has been discussed within distinct but related paradigms that inform KMb. These paradigms include integrated knowledge translation,^12^ co‐production^13^ and participatory research.^14^ The latter, in particular, informs initiatives that recognize the importance of including diverse forms of expertise, and explicitly seeks out lived experience as key information to improve health services and outcomes.

NPOs have an inconsistent, complex relationship with evidence. Their diverse contextual needs make it difficult to build relationships with researchers and/or universities, formalize tacit knowledge and systematize research processes.^15^ Numerous internal and external barriers to engaging with EBPs, and KMb in general, exist for NPOs and their community programmes. Internally, challenges include resource limits in terms of staff, time and financial constraints, as well as a lack of (or perception of lack of) capacity among staff to access and use evidence and evaluation information. External barriers noted include the lack of knowledge‐sharing networks and non‐existent or tenuous relationships with researchers or academic institutions.^16^

### 2.2 Youth engagement

W2A has consistently employed YE as a mechanism to make KMb more effective and resonant to its network of NPOs. In addition to supporting the youth‐serving sector in KMb, W2A has employed YE in all its projects, with the aim of providing a model of YE for other Network members to employ or adapt, based on their needs. Table 1 describes the spectrum of YA involvement in W2A activities.

**Table 1:** Spectrum of YAC involvement in W2A activities

Activity	YA roles and contributions
Knowledge synthesis reports, summaries and checklists	Youth as co‐leads Youth as RAs Integrate YE as cross‐cutting theme and note participatory approach Youth workshops to review each report before finalized Youth designed materials
KMb innovation funding	Youth supported creation of fund guidelines YE as cross‐cutting theme Youth reviewed applications
YAC and GB	Own projects, for example, Mental Health Heroes
W2A events	Youth helped design overall format and approach Youth participate in planning teams Youth lead events as part of youth hosting team Youth co‐facilitate training Youth draft reports
KMb toolkit and mentoring	YE tools in toolkit Offer mentorship on YE co‐led by youth
Academic conferences and publications	Youth lead conference submissions Youth co‐facilitate presentations Youth co‐author journal publications

The current impetus for youth and patient engagement in mental health services, programmes and policies was influenced by the Recovery and Disability Rights movements, 17, 18 participatory research^19^ and community‐development values.^20^ A growing body of scholarship in all these areas affirms that youth have important contributions to make to the design of services and programmes they use, describing the benefits to the youth involved, and to the research processes themselves.^21^, ^22^ A 2017 review of strategies to engage youth in the development of mental health interventions shows that engagement through participation in service development, delivery and evaluation appears to improve maintenance of a recovery focus, and the development of coping and professional skills.^5^ Increasingly, scholars have made recommendations for YE in a mental health research context, describing reciprocal value to both youth and adult researchers involved.^23^ Other scholars have shown that training young people in research processes can improve the delivery of care in mental health‐care settings,^24^ benefit mental health research^25^ and shift local health policies.^26^

Youth engagement is committed to social justice, the foregrounding of previously marginalized perspectives and the practice of continuous reflexivity upon issues of power and privilege. Some scholars have suggested that a genuine commitment to participatory values implies the continual enactment of anti‐oppressive practices that address individual needs and examine structural conditions that lead to oppression in all stages of knowledge creation and mobilization.^27^, ^28^ Demonstrating such application, Stoudt et al^29^ described how self‐reflexive approaches may be applied to situations that involve differentials of power and privilege among youth and adults. Other scholars have addressed the nuances of engaging persons with previous experiences of mental illness and describe the possible accommodations that support work with such individuals.^30^, ^31^ In an examination of patient involvement in quality improvement of services, Armstrong et al^32^ described the distinctive roles that patients can take in mobilizing knowledge between the wider community and practitioners, pointing to the importance of organizational capacities to realize the full potential of engagement. More recently, Heffernan et al^33^ and Hawke et al^23^ described considerations for partnerships between youth and adults within a large mental health services and research organization, including features of the organizational culture that underpin the calibre of engagement outcomes.

## 3 CONTEXT

### 3.1 Wisdom2Action Model

W2A was founded in 2011 with the mission to build a strong, effective and collaborative youth‐serving sector that can better respond to the needs of Canada's most vulnerable young people. Since its creation, young people have been involved in almost every W2A's project. For example, one of W2A's earliest projects was the development of a knowledge synthesis report co‐led by researchers and young people, titled *Working with Children and Youth in Challenging Contexts to Promote Youth Engagement*.^34^ In April 2013, W2A, alongside many young people, held its first network‐wide event, a KMb simulation.^35^

Despite all this early activity, W2A did not have an established youth council during its early stages, due in part to concerns about maintaining such a structure for KMb projects and the scarcity of precedents for such councils. After much discussion and deliberation with youth and adults, Network members were invited to nominate YAs, and the W2A YAC was born.

### 3.2 The W2A YAC

Through its history, youth in the YAC have been nominated to the role by Network member organizations as an intentional response to concerns about capacity (eg the advisory council experience of the youth) and connection (eg youth involvement with other organizations). These two concerns reflected a curiosity about how a youth advisory function would work within a KMb network: it was felt that youth with prior experience in organizations would be better able to consider broad service issues as well as the link between research and evaluation. The W2A has provided support staff to the YAC since its inception. This staff person—usually a youth themselves—supports the YAs in terms of meeting coordination, logistics and project management planning at a national level. A YAC Chair and later two Co‐chairs were named to further support the engagement of this group of young advisors and to represent the Council on the W2A Governance Board.

To date, YAs have been involved in identifying, designing and implementing their own projects, such as *Mental Health Heroes*, a mental health awareness campaign launched in 2014. YAs have helped design project criteria for projects like the KMb Innovation Fund and participated in the adjudication of project proposals to the W2A. YAs have also represented W2A in various settings, such as Parliamentary Committee appearances, liaising with other mental health or youth‐serving organizations and presenting at conferences on behalf of W2A. One of the most visible manifestations of W2A's YE approach has been through its community knowledge‐sharing events. These events invite youth along with all sector actors—including health and social science researchers, service providers, educators, public health professionals and government officials—to consider together how best to support young people on a specific issue, such as sexual violence or providing services to young refugees. YAC participation and leadership in these events appear to have been influential. In post‐event evaluations, the vast majority of participants in W2A events and training sessions have reported an increased awareness of how YE could contribute to policy and programme development in their own organizations as a result of their experiences with the W2A YA and adult team. Table 1 provides an overview of activities involving the YAC.

## 4 EVALUATION METHODS

In the fall of 2017, W2A conducted a participatory evaluation involving members of the YAC, W2A's core team and W2A's Board. The goal of this process was to articulate the story of W2A's YE and to collectively envision next steps. An independent evaluator (Canas) was hired to facilitate the process. In keeping with the tenets of participatory evaluation,^36^ youth in the YAC joined in collaborative discussions that simultaneously trained them on the goals of this type of evaluation and led them to articulate the kinds of questions and outcomes they found most relevant.

We set out to understand and assess the effectiveness of YAC engagement in two ways: (a) in relationship with the values and aims of YE and (b) in relationship with the W2A's stated purpose to support the youth‐serving sector. Questions to explore this included the following: (a) How has Youth Council engagement impacted YAs, staff and the organization? (b) Can we link changes in culture, leadership, vision, strategy to the presence of the Youth Council? (c) What is the W2A's role in the development of its YAs as part of the youth‐serving sector? and (d) how well are we carrying forward the voices of particular populations of youth?

Five W2A YAs participated in individual, confidential interviews, which were recorded, transcribed and coded for emerging themes. Textual sources of data used included a conference presentation created and delivered by the YAC in fall 2017 and two web‐based publications released as a result of the Network's impact evaluation in 2015.^37^ A synthesis of themes from these interviews was shared with the YAs for clarification and input. The evaluator then interviewed one W2A staff and two Board members. Emerging themes from these interviews were incorporated into the previous analysis and brought back to the YAs for further discussion and input.

A second round of interviews was then conducted with the YAs, in order to discuss and deepen data collected so far. As a last participatory step, a draft version of this report was distributed among all persons who participated in interviews, and their perspectives were used to further refine the final report. This article is a result of collaborative synthesis of the report, and further reflection, on the part of the authors. Pseudonyms have been used in this publication to preserve anonymity. The data that support the findings of this article are available from the corresponding author upon reasonable request.

## 5 RESULTS

In the following paragraphs, the themes derived from this evaluation are described in relation to the particular areas of focus and concern expressed by the YAs.

### 5.1 YAC engagement impact upon youth, adults and the organization

All YAs in this programme reported positive individual outcomes in their sense of self‐efficacy and identity as a result of participation as advisors. The YAs described feeling heard and supported at the organizational level when they interact with W2A staff. Richard, a YA, said: “I feel totally engaged with the Network, and quite valued … I feel supported as a youth, and that my agenda is supported.” Engagement has increased partnerships and networks, as well as capacities, for these youth. In describing how their engagement has advanced their advocacy and public speaking skills, YA Emma said: “I feel the Network has been really supportive when someone has proposed a conference or submitting an abstract … supporting youth leadership.” At the same time, this young advisor recognized how difficult it is to track outcomes of engagement upon an individual. Emma said: “I feel it would be hard to map the ways that I've grown because my experiences have been connected with so many other things.”

#### 5.1.1 Mutuality of gain

Mutuality, the fact that not only the YAs but also the adults gain from engagement, emerged as an important finding. Hugh, a YA, said: “Engagement that works best is one where everyone – including adults and professionals – feel like they have gained from the experience.” Joan, a W2A team member, described the gains to the organization and their work as follows: “New ideas for priorities, activities and projects… feedback on other things we might come up with … a huge amount of capacity in community‐building, events‐design and facilitation. The benefits have been huge in terms of the different types of work that people in the YAC have taken on, as a community and, also, individually.”

### 5.2 Engagement inputs, facilitators and challenges

Inputs of YE identified consistently as part of the processes at W2A included mentorship, skills building, financial and reputational resources, and clear communication and expectations. These inputs were seen to generate effective engagement activities that contribute to the Network's mission to improve the sector and, hence, youth well‐being. Additionally, both youth and adult participants in this evaluation identified facilitators and challenges, including flexibility, supportive mentorship, retention, professional development and the sustainability of engagement efforts.

#### 5.2.1 Flexibility

Youth and adults spoke of the need to factor in the changing circumstances of young people’s lives, as well as fluctuations in their wellness (defined to include physical, mental and financial health) as a determining factor of their engagement. This is an ongoing challenge in the context of a national youth council. Describing how difficult it can be for youth to prioritize this kind of role, Laura, a YA, said: “It can be confusing because it's not an everyday commitment. Meeting over the Internet is not the most engaging thing to do for a lot of people. It's a lot harder to maintain the focus.”

#### 5.2.2 Mentorship

Participants recognized that YAs benefit from supportive mentorship, while the adults in both the Board and the organization shared that time and resources needed to mentor youth were scarce due to organizational realities. Describing some of the time resources necessary to provide supportive mentorship, Joan said: “A lot of it is the flexibility to talk, brief and debrief, build knowledge when and if youth are available. Being there for them. Then, schedules change a lot for them, and require last‐minute changes, or the ability for staff to fill in when something falls through for an advisor.” From a Governance Board level, supportive mentorship was perceived as a separate way of operating from the usual processes of that group. Steven, a Board member, said: “If mentorship is something that we seek to do intentionally, we will need to include that in the terms of reference with current members, and make it a part of the commitment. Different skill‐sets may also be needed.”

#### 5.2.3 Retention

Adult participants noted that keeping youth engaged is subject to the same challenges as retaining volunteers in board and volunteer management. Joan described the tasks involved were “sustaining people's interest, adapting to everyone's schedules, and securing regular meeting attendance and prompt email communications.” Participants agreed that facilitators of effective board and volunteer management would also benefit YE. These included supported and increased communication across YAs and from the organization to the YAC, as well as policies that support the YAC in its current activities. Laura described the need for better documentation and tracking of processes for the YAC: “I wonder if some of the stuff needs to be written in a policy. If there's a conference you want to go to, this is the way you can pitch to go to a conference. Some people will put their name forward, but others may not know that it's an option (to propose a conference).”

#### 5.2.4 Professional development

An important challenge to sustainable engagement centred on the professional development of youth and their growing need for market‐level remuneration. All participants expressed the recognition that volunteer and semi‐volunteer activities are not sustainable for young people, a message that the Network has mobilized among NPOs as a best practice. Emma YA said: “We are mentoring all these organizations and we're already telling them that they shouldn't ask for youth involvement for free. We're telling them that they should be paying people, but sometimes paying them a living wage for where they live is still sometimes coming up short.” In this YAC, tensions existed between young people's need for growth (economic and professional) and their current capacity in the context of YAC activities. Though their skill sets were evolving, in part due to their involvement with the Network, their current level of professional performance and ability to deliver on their responsibilities was not yet at market level in the KMb arena. A value‐based approach to engagement requires both sensitivity to each YA's individual capacities and strengths, and the recognition that the organization must remain sustainable by remunerating appropriately for the quality of work delivered.

#### 5.2.5 Organizational sustainability

Because all of the challenges and facilitators described require organizational resources, the commitment to deliver on the values of YE represents an investment and challenge of sustainability for W2A. YAs felt that the mentorship and capacity building of YAs is a means of strengthening the youth‐serving sector itself. As such, the investments of YE are wholly in line with the organizational mission and vision. This stance was supported by the adult participants. Mark, a Board member, stated: “Particular engagement tactics may be different for youth, and may require more investment in capacity building … But it is up to the organization to articulate the rationale and resource‐ allocation approach to address this particular way of delivering on its values.”

### 5.3 The diversity and quality of youth‐perspective representation

All youth and adults interviewed noted the use of effective scaffolding and supportive techniques in the way W2A events are developed, with the net effect that youth at different levels of advisory role skills are able to participate in W2A Network and KMb events. However, consistently, the YAs described a wish that they could provide more direct input in the vision and strategic development of the W2A. Emma said: “Racialized youth are on the YAC, and we're doing the on‐the‐ground work with the youth sector, but are not necessarily reflected in the decision‐making or future‐setting of the organization … The youth don't only need to be on the YAC, they could be involved at other levels of the organization.”

The YAs want to ensure that this level of diversity and quality of youth representation is present in all W2A activities, in particular in the governance and strategic operations of the Network. They described feeling that there is less communication with and impact upon Board decisions and W2A direction. Challenges centred on the fluidity of communication between YAs on the Board and the rest of the Council, as well as on the actual language and conduct of Board meetings. YAs asked for more training in specific areas that would support them to contribute to the strategic direction of W2A (eg on social‐enterprise models).

This finding also emerged when interviewing members of the W2A Governance Board. In discussing the inclusion of youth perspectives in Governance Board meetings, they noted that often the discussions moved fast and that the YAs may need time and clarification to be able to participate fully. While the YAs expressed that this challenge of participation could be addressed by using more explicit anti‐oppression lens in Board operations, Board members asked to receive training in participatory approaches to engaging diverse perspectives, as a means to ensure that engagement is meaningful and sustainable. Steve said: “It should not be assumed that the Board members know what YE and anti‐oppression practices are, or how they work.”

## 6 DISCUSSION AND IMPLICATIONS

### 6.1 Participatory evaluation as a tool for organizational self‐reflection

This evaluation process, in its question generation, analysis and iterative consultation, adhered to the participatory tenets of foregrounding the voices of persons previously excluded as knowers or experts.^3^, ^19^ The history of involvement between W2A and its YAs—as well as the optimism and openness to change relayed through the interviews with both youth and adults—is congruent observations from scholars in the field of YE. For example, Roach, Wurta and Ross have noted young people's willingness to embrace adult‐provided structures through the processes of change needed in many organizations that deal with youth.^38^ The perspectives shared by this group of YAs and W2A adults show that it is possible to maintain simultaneously critical and committed positions to YE.

This methodological approach to evaluation of organizational processes is not without its challenges. Some of the issues and questions that arose were difficult to discuss and underscored how, even in organizations where the values and culture are oriented towards youth well‐being, existing power and social‐location differentials exist between adults, the institutions they represent and the young people who hope one day to be employed by such institutions. The role of the organizational culture in supporting this type of deep and sensitive inquiry cannot be overstated and merits further investigation. This finding aligns with Heffernan et al and Hawke et al, who describe the self‐reflexive work needed in such partnerships within a research and service context.^23^, ^33^

At its broadest level, this critical self‐reflection on the part of the W2A and its YAC served to highlight how structural dimensions of engagement play out in the interactions between youth and a not‐for‐profit organization. Participatory evaluation supported youth and adults to articulate the perceived impacts of YAC engagement and to highlight the kinds of barriers and facilitators related to sustainable engagement. Through this focus, this article begins to address the question in scholarly discussions of engagement, namely whether “well‐meaning adults concerned with genuine YE [can] erase the naturalized boundaries — often unacknowledged — between youth and adults created and maintained in our cultures, institutions and discourses”.^39^

The approach of engaging an external consultant may have supported the openness of the discussions undertaken and the YA's and adults’ willingness to reflect critically upon their experiences. The role of value‐aligned adults in facilitating processes of knowledge generation is a core consideration for many YPAR researchers.^29^, ^39^ The skill sets suited to address the nuances of participation with youth may be present among a range of practitioners in health and social services, as well as in education and KMb. NPOs seeking to engage YAs in their knowledge generation and evaluation processes may benefit from reflecting on the culture and internal capacity of their organizations before undertaking such processes.

### 6.2 Benefits of YE for youth and organizations

YAs reported having positive experiences, noting that engagement has increased partnerships, capacities for communication and self‐advocacy for them. This supports the scholarly consensus that YE—when effective—is of benefit to the young people's development, sense of efficacy and identity, and feelings of social inclusion.^21^, ^27^

W2A staff and Board members feel they have gained in terms of new and diverse perspectives, innovative thinking and contextually relevant knowledge as a result of engagement with the YAs. The mutuality of gain among YAs, W2A and other W2A Network stakeholders makes the YAC, collectively, a viable and necessary recipient of W2A services and resources. These positive outcomes underscore benefits to organizations when youth are engaged; similar outcomes have been found in youth‐engaged processes of knowledge generation^25^, ^27^ and policy development for the youth‐serving sector.^26^, ^35^

A significant finding from this process is that the YAs position themselves as part of the youth‐serving sector, rather than solely recipients of services. This is an important distinction that aligns with YE and participatory values, which see youth as active agents of change and both subjects and architects of research.^19^ This stance on the part of youth, and the adults who champion their engagement, interacts in a crucial way with the organization's mission to strengthen the youth‐serving sector, creating a built‐in rationale for W2A to continue its investments in youth mentorship and engagement. NPOs contemplating YE may do well in considering how a YAC, or other investments in YE, relates to their organizational mission and values, particularly under an equity orientation.

### 6.3 The need to better understand impacts

This process underscored the need for ongoing evaluation efforts at the broader W2A level, to better understand how the Network is supporting NPOs through all of its activities, including the inclusion of YA perspectives. YAs feel heard by staff within the organization and feel that their input into W2A events is heard and implemented; however, they are uncertain whether their impact extends to the strategic vision of the organization or the broader Network members and sector. This concern aligns with known challenges in measuring the impact of both engagement^14^ and KMb activities.^11^, ^12^

### 6.4 Directions for growth

The directions for growth identified in the experiences of this YAC and W2A are consistent with the changing needs of both youth and the organization, particularly given the extended nature of their engagement. In an examination of mid‐ and late‐phase research and partnership, Lincoln, Borg and Delman described the increased levels of training and negotiation needed among community and academic partners in order to reconcile changing priorities and incentives, competing timelines and the equitable allocation of resources.^25^ In the context of W2A, some YAs asked for more frequent and supported communications and noted training needs that would allow them, and an increasingly diverse group of YAs, to participate more meaningfully at governance and strategic levels of the organization.

Board members mentioned their interest in learning more about YE and the anti‐oppression practices that inform it, as part of making the culture and values of W2A more explicit. Scholars in the area of positive youth and community development have long advocated for this type of organizational policy and adult‐partner training as a core element of effective youth‐adult partnerships.^40^ A self‐reflexive stance towards the professional and egalitarian aspects of working together is called for in the context of partnership with persons with lived experience of mental health challenges.^41^ In the case of W2A and the NPOs in its network, the clear training directions requested by YAs and Board members constitute a direct opportunity for W2A and its stakeholders (including funders) to advance their commitments to the youth‐serving sector.

Another area of training highlighted by the YAs in this process relates how to sustain engagement across different time zones and geographic areas. The pan‐Canadian nature of this youth council makes it sometimes difficult for youth to participate. Managing a remote, national‐level role of intermittent activity can be challenging to youth given the many demands of (and sometimes multiple jobs required to sustain) living. Though scholarly writings in YE often mention challenges relating youth schedules and competing life priorities,^42^ direct guidance that addresses the contextual needs of both youth and national‐level NPOs is scarce. W2A's own learning on how to manage its pan‐Canadian network is a best practice that merits sharing with its own YAC and with other organizations of national scope.

Increasing the YA's capacities to expand their contributions as members of the youth‐serving sector is both an outcome of engagement and an important technique for its longevity. In the context of engaging youth who may have had experiences of ill health/mental health, the commitment to support youths’ professional development occurs alongside fluctuations in their well‐being. W2A's commitment to being flexible, allowing YAs multiple opportunities to step in and step out of engagement, has been an important facilitator of professional development for the YAs. W2A staff and Board members described this flexibility as “walking the walk” of its values. The particular techniques employed, however, are consistent with work accommodations described in research of service organizations.^31^ The costs of such flexibility tend to be indirect and relate to extra supervision or training, flexible scheduling and modifying job duties.

The YAs interviewed reported that they felt they had evolved in their educational and professional trajectories, and could now contribute at a different level as professionals in the sector. Their interest in contribution to the strategic development of the organization, including its vision forward, is consistent with other findings regarding the spectrum of patient engagement in health‐care organizations. Carman et al found that engagement in organizational design and governance is a logical progression for individuals who had developed in roles as advisors at the level of care. Activities listed at this level of partnership included participation in quality‐improvement teams, taking part in hiring decisions, and development and training of staff.^30^

From the organizational perspective, the supportive mentorship and capacity building involved in the retention and professional development of YAs is an investment that ensures W2A delivers on its values and visions. However, such investments may also challenge the sustainability of an organization and must be part of its articulated strategies for funding.

Whether or not youth perceive W2A activities as “employment” affects their level of engagement, including their response time and quality of input into decisions and processes. Some youth in this Council have outlived their time as volunteers and are feeling less motivated to engage. Others would like to get better remuneration for their facilitation roles. At the same time, some of the youth in the current Council may not have the full range of skills and competencies that put them on par with professionals in the facilitation and/or event coordination market. This is a significant challenge in all engagement processes and requires frank, ongoing conversations between the organization and the YAs, in order to determine everyone's expectations. The issue of appropriate remuneration is emergent in scholarly discussions of YE. Indeed, some researchers examining YE in various contexts address the importance of paid youth roles as a necessary recognition of  young people's time and contributions.^2^, ^3^, ^23^, ^33^ However, no studies retrieved addressed instances of youth payment that exceed the honoraria set under Research and Review Ethics Board guidelines, which are kept purposefully low to avoid the possibility of coercion, and do not correspond to the market‐level facilitation rates. An important mechanism to advance this practice will be to articulate, to funders and other partners, the value of training and of equity‐oriented remuneration of YAs as they transition into professional roles.

## 7 CONCLUSION

In this article, we have shown that, to date, YAs to the W2A Network see themselves within their various locations—social, cultural, ethnic and professional, to name a few—as contributing members to the youth‐serving sector. YE in W2A's activities results in clear gains for both the youth and the organization, including the growth of individual networks, mentorship opportunities and capacity for youth, and increased networks and diverse perspectives for the organization. The personal and professional development of YAs constitutes another W2A contribution to strengthening the sector and makes YE in this context a worthwhile investment.

The tension in this YAC and much other YE work lies in the balance between mentoring and supporting youth as part of a population that have traditionally been excluded, and ensuring ongoing genuine engagement within the management and governance of the organization.

Supporting YE requires organizational resources in terms of policies, processes and finances, with a constant vigilance to ensure management and governance processes are assessed as to how they are integrating YE. For national organizations like W2A, it is important to fund YAC activities, such as in‐person meetings, facilitate ongoing online communication and provide coordinating staff to support to the YAC to facilitate meetings and project activities. Lastly, a challenge in engagement, and echoed in the field of KMb overall, is helping the YAs to see the impact of their work on the sector.

These findings and recommendations hold the potential to support engagement in health service and other organizations that serve youth. Findings may also be of relevance to funders, in order to better understand the impact and rationale for youth engagement in many types of projects, including KMb.

## CONFLICT OF INTEREST

The authors declare no conflict of interest.

## Data Availability

The data that support the findings of this article are available from the corresponding author upon reasonable request.
